# Conspecific "gaze following" in bottlenose dolphins

**DOI:** 10.1007/s10071-022-01665-x

**Published:** 2022-09-05

**Authors:** Christine M. Johnson, Christina Ruiz-Mendoza, Clara Schoenbeck

**Affiliations:** 1 Department of Cognitive Science, University of California, Gilman Drive, La Jolla, San Diego, 9500 USA; 2 Marine Science Program, Scripps Institution of Oceanography, UCSD, Kennel Way, La Jolla, San Diego, CA 8622 USA

**Keywords:** Gaze following, Bottlenose dolphins, Conspecifics

## Abstract

"Gaze following"—when one individual witnesses another shift its orientation, and then re-orients in the same direction—has been observed in a wide range of species. Related work with dolphins has to date focused on human–dolphin interactions. In this conspecific study, we examined a group of dolphins orienting, in passing, to gateways between their pools, as opportunities for witnesses to demonstrate "gaze following". Seven bottlenose dolphins were synchronously videotaped on six underwater cameras, for 21 h over three days, and the recordings analyzed by trained observers. The identities of all animals present, their partner state, and whether and to what degree they had altered their access to the gate (e.g., from Monocular to Binocular, or Binocular to *Visio-Echoic*) was recorded. Compared to animals that did not witness such a change, witnesses of an increase in access by another dolphin were significantly more likely to also act to increase their own access. We observed 460 such cases of "gaze following" in these animals. Dolphins who were partnered (showed sustained swimming within 1 body length) were significantly more likely, than non-partnered animals, to "gaze follow". Dolphins also showed a significant tendency toward matching the kind of access they observed. No significant difference was found in the presence of animals in the back pools, during changes in orientation that were followed, versus in those that were not. These findings support adding bottlenose dolphins to the growing list of species that display conspecific "gaze following".

## Introduction

### Gaze following in nonhumans

"Gaze following" occurs when one individual observes another shift its sensory orientation to a particular target or vicinity, and then the witness shifts its own orientation in that same direction (Scaife and Bruner 1975; Tomasello et al. 1998; Emery 2000; for overview, see Shepherd 2010). This can provide access to critical environmental information that the witness might otherwise have missed, as well as cues to the other individual's likely subsequent behavior.

In vertebrate species, sensors—such as eyes, ears, olfactory sensors, tactile detectors such as whiskers, taste receptors in the mouth, etc.—tend to be located on the leading edge of the animal's body (Webster and Webster 1974). As animals generally orient their sensors in the direction they are likely to move, or to which target they are most likely to respond, such shifts in orientation are reliable predictors of behavior. These regularities are available to others, in social and physical interactions, and can be exploited in a variety of contexts (for reviews, see Emery 2000; Itakura 2004; Zuberbühler 2008). In many species, for example, gaze following can promote the detection and avoidance of predators, coordinate group travel, scaffold young animals' learning to locate, identify, and process foods, etc. In a social context, it can play a role in discriminating the possession or contestation of resources, in anticipating and interpreting interactions between others, and in otherwise negotiating the complexities of multi-party engagement. In humans, of course, who also show these skills (see Moore 2008; Flom et al. 2017), this extends to gaze interactions facilitating the coordination of complex social practices, such as the sharing of language and culture (e.g., Brooks and Meltzoff 2015; Shepherd 2010).

Given how much it can contribute to fundamental skills like foraging, defense, and social coordination, it comes as little surprise that gaze following has been observed in a number of nonhuman species. Many of these are highly social, including primates (Emery et al. 1997; Tomasello et al. 1998; Brauer et al. 2005; For reviews, see Johnson and Karin-D'Arcy 2006; Rosati and Hare 2009;), dogs (Miklösi et al. 1998; Hare and Tomasello 1999; Wallis et al. 2015), corvids (Bugnyar et al. 2004; Schmidt et al. 2011), horses (Wathan and McComb, 2014) and other herd-forming taxa (e.g., Kaminski et al. 2005; Schaffer et al. 2020). However, as researchers widen the scope of these inquiries, it is becoming apparent that gaze following can also be observed in what are more typically considered 'asocial' species, such as the leopard gecko (Simpson and O'Hara 2019) and the red-footed tortoise (Wilkinson et al. 2010). Furthermore, cross-species interactions, such as between resource competitors, or predator and prey, may also be mediated by attending to the orientation of others (Caro 2005). To date, research on inter-species gaze following focuses primarily on human–animal interactions (more on this below).

While it is becoming apparent that the fundamental practice of gaze following is probably a very wide-spread phenomenon, it is also likely that the underlying cognitive mechanisms involved vary across species. While, even in humans, gaze following sometimes appears to be a rapid, reflexive response (Friesen and Kingstone 1998; Teufel et al. 2010) it can also play a role in more sophisticated cognitive processes, such as vocabulary development (e.g., Tomasello and Farrar 1986; Morales et al. 1998;), reading intentions during collaboration (Huang et al. 2015), and making attributions of knowledge to others (Freire et al. 2004; Kaminski et al. 2008; Whiten 2013). Thus, while evidence of gaze following, alone, is not sufficient to make claims for "theory of mind" in any species, it is likely a necessary prerequisite for that more complex ability.

### Bottlenose dolphins

Bottlenose dolphins (*Tursiops* sp.) are inter-dependent group-living mammals, with complex social structures, including coalition formation and collaborative hunting (Wells et al. 1980; Connor et al. 2000; Connor et al. 2001). As such, we might well expect them to be good candidates for "gaze following". However, no study has yet examined this practice between conspecifics. This paper aims to redress this by focusing entirely on dolphin–dolphin interactions.

There has, however, been related work on interactions between dolphins and humans (for review, see Pack and Herman 2006). For example, Tschudin et al. (2001), and Pack and Herman (2004) found that bottlenose dolphins would correctly follow human head-turns (left or right) to one of two choice objects. The latter study also found that an "eyes-only" (head stationary, eyes shifted to one side) cue was ineffective in this task. Pack and Herman (2007) report that the same dolphins had difficulty determining, based on (upward/downward) head-tilts in a human, whether the target object was the proximal or the distal one, along the same line of sight. One dolphin could, however, judge the difference in the angle of outstretched-arm points, well enough to select the appropriate object (see also Herman et al. 1999). Dolphins' training history may well play a role in how much they attend to human hands versus the human face. Tomonaga et al. (2010), for instance, found that their dolphins would still reliably respond to a trainer's hand signals, even if the trainer's heads were turned away or were hidden from sight. However, the trainers in these studies also typically report that their animals are required to begin a trial, in a stationary position, by making "eye contact" with them. While such inter-species interactions can be instructive, it seems that if a sensitivity, and responsiveness, to a change in sensory orientation does naturally emerge in these animals, it can be expected to be tuned to detect and use such changes by conspecifics (see Kano and Call 2014).

### Species-specific constraints

It is important to consider, in whichever species is being studied, both the constraints on that animal's sensory abilities, as well as the nature of the attentional behaviors that they display. Primates, for example, unlike many mammals, are unable to orient their pinna (external ears) toward a particular sound source, and so that display is unavailable to them. In contrast, primate hands, freer from the locomotor or support functions typically served by the forelimbs, provide many displays of haptic attention. Primate hands are sensitive and dexterous, and through hand–eye coordination, are the main interface between that animal and its world (see Freedman and Sparks 1997). Like gaze, hands target certain objects, such as food or conspecifics, and thus provide predictive patterns of orientation and approach that other animals can observe. Thus, tracking the effects of both hand and head movements would be an appropriate approach to studying "attention following" in that taxon (for examples, see Anderson et al. 1996; Itakura 2004).

"Gaze following" does certainly occur in primates (e.g., Tomasello et al. 1998; Ferrari et al. 2000; Okamoto-Barth et al. 2007). With their forward-facing eyes, primates have a visual field that extends only about 90° either side of the midline, and about 50° above, and 60° below, the point of focus (Kaas and Collins 2003). As a result, access to the remainder of the visual field requires active head and/or body turning, making such movements common and potentially informative. In addition, especially depending on the witness's perspective, eye and head direction are often redundant cues. While some primates have occasionally been shown to be able to use eye direction alone as a cue for gaze following (e.g., Povinelli and Eddy 1996; Ferrari et al. 2000), given that moves of the larger head are much easier to detect, most primates are more proficient with head-turning as the cue (Tomasello et al 2007; Rosati and Hare 2009).

**Fig. 1:**
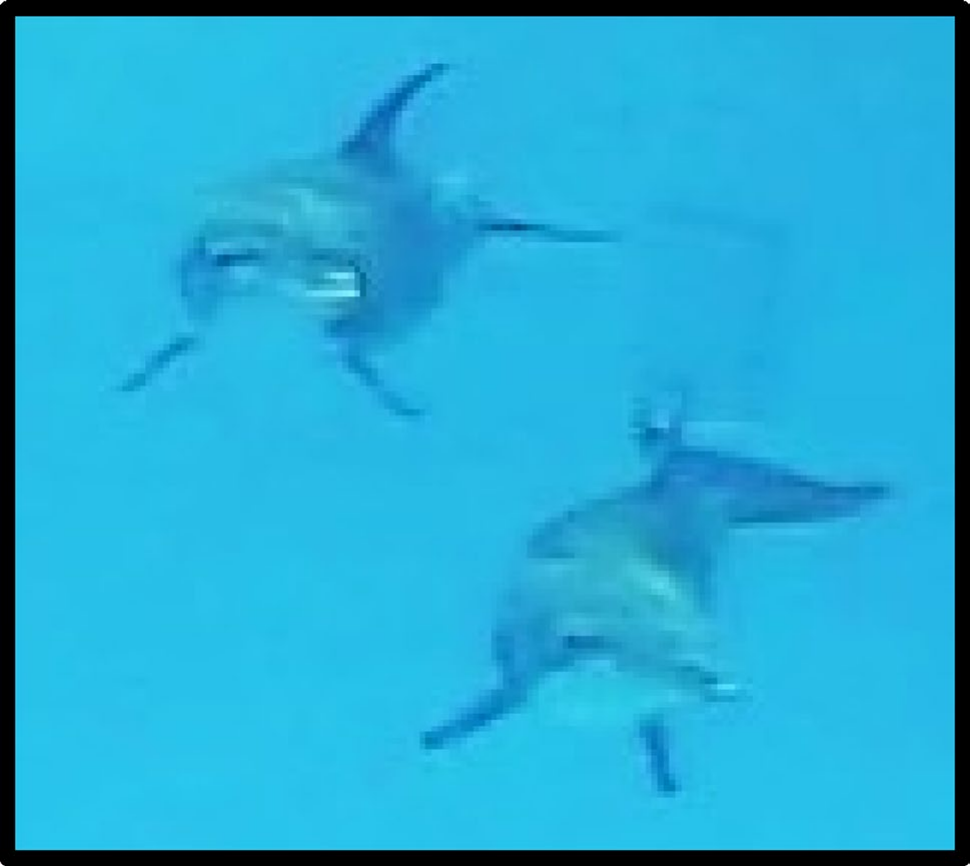
Head Turns

**Fig. 2:**
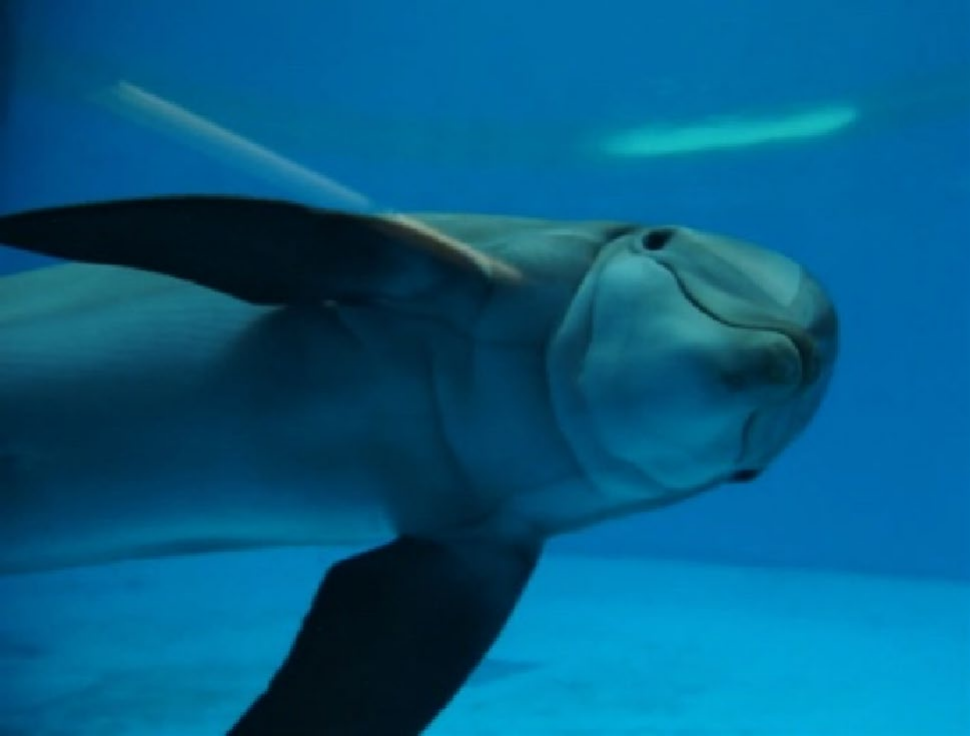
Binocular vision below and forward of head

Dolphins show both similarities and differences in their attentional behavior, compared to primates. Due to hydrodynamic streamlining, dolphins are deprived of many of the mobile facial features that play such an important role in mammalian communication (Norris and Dohl 1980). Nonetheless, their primary sensors can also serve as sources of information for others. In most genera, including the bottlenose dolphins studied here, their dark eyes are further demarcated by a surround of pigmentation, much darker than the lighter grays of the nearby skin (see Figs. 1 and 2). Such high contrast is likely to make eyes salient to other dolphins, whose vision, while not including color, is particularly sensitive to contrast (Madsen and Herman 1980; Herman et al 1989).

Each eye is laterally positioned, typically providing the animal with over 200° of visual access to its surroundings (see Hanke and Erdsack 2015). Areas not typically covered include the area above and in front of their head, and directly behind their back. Dolphins are also capable of protruding their eyes, and swiveling them to get a better view either ahead or behind, and can move each eye independently. While their cervical vertebrate are fused, limiting their head movement (Thewissen et al 2009), the dolphins are still capable of turning, arching or bowing their heads, and often do so to scan their environment (see Fig. 1).

Dolphins have limited (20–30 degrees, per Supin et al. 2001) binocular vision, extending principally in front of and just below their heads, and it is only from that area that two eyes can be seen on the animal at once (see Fig. 2). In the wild, seeing down from above no doubt enables them to watch for predators and prey, as well as to monitor the behavior of conspecifics. Note too, that since mating in these animals occurs ventrum to ventrum, and tilting one's bright underside toward another can be a solicitous behavior (e.g., Johnson and Norris 1986), binocular access may be typical of such interactions. All of the above suggest that eyes are available to serve as predictive cues, correlating with the animal's direction of movement, head/body orientation, and various types of activity.

Another important sensory system in dolphins is echolocation (Au 1993; Cranford, Amundin and Krysl 2015). A series of pulsive, wide-band "clicks" is generated in the nasal areas of the dolphins forehead, and projected out through a lens-like "melon" that focuses the output into a narrow beam. This echolocation beam forms a rough cone, emanating from the animal's forehead, typically extending about 10° either side of the midline, with both peak frequency and power quickly decreasing with distance from the central vector (see Branstetter et al. 2012). The animals receive the returning echoes from their sonar (along with whatever ambient sound impinges on them) through fat channels in their throat and lower jaw (Brill 1988). These channels carry the sound to their inner-ear complexes, located at the ventral-posterior sides of their head. (Cranford, Amundin and Krysl, 2015). Note that, while they cannot see the main area occupied by their echolocation beam, the forward vision they do bring to these events—such as while chasing down prey—is binocular.

Dolphins are also capable of "steering" their echolocation beam, shifting its orientation even without moving their heads. For example, Moore et al. (2008) report that a bottlenose dolphin, with its head in a fixed position at an underwater bite-plate, could detect objects set 18° off the midline. Working with a younger animal, and a higher resolution hydrophone array, Starkhammar et al. (2011) found an even greater horizontal displacement, with a fixed-position animal detecting objects 28° either side of center. Data on the frequency and amplitude profile of this animal's echolocation further suggested that this may have involved simultaneously generating clicks at both sound sources in the animal's head. Dolphins have independent control of these bilateral foci of vibrating tissue, and are capable of activating only one (typically on the right side for echolocation), or both, or even producing clicks at one and whistles at the other (Madsen et al. 2013). Other research suggests that, while chasing down prey, dolphins can broaden their beam width, when they get close, perhaps to decrease the risk of a sudden escape (Jensen et al. 2015).

Research has verified that dolphins are also capable of "listening in" to the returning echoes of others—often called "eavesdropping"—when they are aligned side by side. For example, in an underwater match-to-sample task (Xitco and Roitblat 1996), one dolphin was free to echolocate on the sample object, suspended behind a visual barrier. The eavesdropping dolphin was parallel to the first, but positioned at a bite-plate at the surface, such that its jaw was underwater, allowing it to hear, but its melon was above the waterline, preventing it from echolocating. Nonetheless, based only on the echoes from the other animal's sonar, the eavesdropper was often able to select the matching alternative, although its performance was somewhat degraded. In the wild, dolphins traveling together in a tight, parallel formation are more likely than those in a loose, asymmetrical group to have only one or a few dolphins echolocating at any one time (Gotz Verfuss and Schnitzler 2006). This suggests that these animals may exploit eavesdropping while searching for food, to reduce individual energy expenditure. Note that the forward-projected echolocation beam is so tightly directional that one animal swimming beside another, while well positioned to hear the echoes, would at best have minimal access to the outgoing clicks. Thus, the similar alignment of their heads not only gives them access to echoes, but allows them to experience, and observe in others, the functional significance of such co-orientation. This is true of the dolphins since infancy, since they spend the first year of their lives tightly beside their mothers (McBride and Kritzler 1951; Gubbins et al. 2006), in the ideal position to listen-in and learn.

In sum, head or body movements that alter the orientation of a dolphin's eyes and/or forehead toward a target, can be predictive of various types of access to that target. As a result, "gaze following" merits the scare quotes surrounding it in this paper, since while such changes in a dolphin's orientation *might* involve following gaze, many instances may involve "beam following" instead. In this study, we track not only whether a witness dolphin observed, and then reproduced, another dolphin's change in orientation toward a particular target, but also whether that change was likely to have provided the animals with monocular, binocular, or visio-echoic access to the target.

## Methods

### Subjects

The subjects in this study were 7 bottlenose dolphins (*Tursiops truncatus*), in residence at the Brookfield Zoo. This included 3 adults (1 male and 2 females), 3 sub-adults (1 male and 2 females), and one juvenile female (see Table 1). Three of these subjects are related: one adult is the mother of the juvenile and of one of the sub-adult females.

**Table 1:** Individual differences in gaze following performance

ID	"Gaze following" events	Witness events, did not "gaze follow"	Tendency to "gaze follow"
Adult male	79	111	*X*^2^ = 46.772, df = 1, *p*-value < .001
Adult female (mother)	153	99	*X*^2^ = 170.39, df = 1, *p*-value < .001
Adult female	68	88	*X*^2^ = 31.835, df = 1, *p*-value < .001
Sub-adult male	49	41	*X*^2^ = 37.811, df = 1, *p*-value < .001
Sub-adult female (daughter)	49	60	*X*^2^ = 35.535, df = 1, *p*-value < .001
Sub-adult female*	7	20	*X*^2^ = 4.4528, df = 1, *p*-value = 0.03484*
Juvenile female (daughter)	55	91	*X*^2^ = 21.771, df = 1, *p*-value < .001

The first column shows the number of gaze following events observed for each individual. The second shows how often each witnessed a Turn To by another, but did not gaze follow. The third column shows the values of Chi-Square tests of Independence, with Bonferroni Corrections, concerning each individual's tendency to gaze follow after witnessing another Turn To a gate. Only the unrelated sub-adult female*, with the smallest number of observations, failed to reach significance

### Setting

**Fig. 3:**
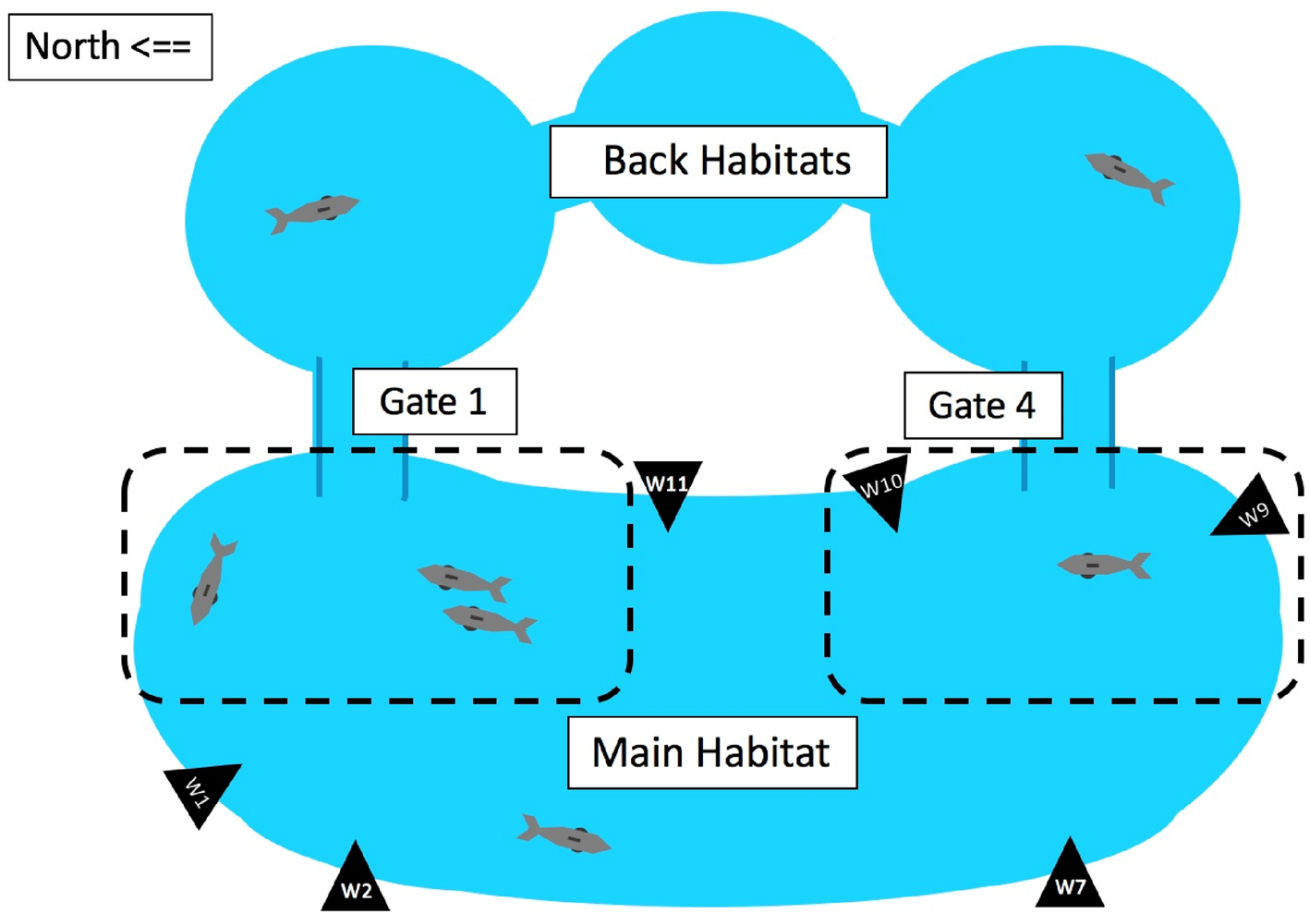
Dolphin facility. All observations were made in the Main Habitat. Gate 1 and Gate 2 are the gates through which animals in the main habitat can direct their attention into the back habitats. The black triangles indicate underwater cameras. Video of passes by Gate 1 were shot from windows W1, W2, and W11, and by Gate 4 at W7, W9, and W10. The hatched lines encircle the "vicinity" of each gate, the areas in which gate passes were assessed

The facility in which the subjects were housed consisted of four inter-connected habitats (see Fig. 3). All observations for this study were conducted in the (110’ X 40’ X 25’) main habitat. Two passageways (10’ X 6’ X 6’)—called “Gates”—one at either end of the main habitat, each connect to a different back habitat (see G1 & G4 in Fig. 3). Between formal training sessions, the animals were generally allowed to move throughout this enclosure, and animals in the main area often appeared to adjust their swim patterns to gain perceptual access, through the gates, to the back habitats. All video analysis was focused on this time between training sessions, when no significant human presence was involved.

### Procedure

Video was recorded using LG Dome Security Digital Video cameras (Model CD14WDR) fixed at six underwater-viewing windows around the main habitat (see triangles in Fig. 3). A total of 42 h of video from the main habitat were collected and analyzed. For each gate, three cameras recorded 21 h of synchronized footage, providing multiple, underwater views of the animals’ behavior near the two gates. Cameras at Windows 1, 2 & 11 were used to assess activity around Gate 1, and Cameras at Windows 7, 9 & 10 were used to assess activity around Gate 4. These videos, shot over three days, February 2013, between 8 am and 5 pm, were then analyzed for any changes in perceptual access through those gates by animals in the main habitat.

As described above, given the dolphins’ sensory morphology, different positions in the water relative to the gates afforded different types of perceptual access. Given its laterally placed eyes, a dolphin swimming upright, directly past a gate, would have monocular visual access to the back habitat. In contrast, given that binocular vision is limited in these animals to the area directly in front of, and largely below, their heads, the dolphin would need to tilt its ventrum to the gate as it passed to gain binocular access. This could also be achieved by the animal rising up from below the gate, with its ventrum towards the wall. Since the dolphins’ echolocation beam emanates forward from their forehead, with the majority of energy confined to a narrow cone, if the dolphins turned their heads or bodies such that this beam could be projected directly through the gate, they were coded as having both visual and echoic access. If an animal passed below the gate, it would be recorded as gaining no access. As a result, all passes by a gate were coded as providing *None, Monocular, Binocular*, or *Visio-Echoic* access.

The data for each gate were collected separately, based on the three views the cameras afforded to each gate. A scene began when at least two animals entered the vicinity of a gate. "Vicinity" was defined as the eastern half (gate side) of the main habitat, including either the northern third (near Gate 1), or southern third (near Gate 4) of that habitat (see hatch-marked areas on Fig. 3). In particular, a dolphin would need to be observed swimming north of Window 11, or crossing Window 10, to be included in a scene for Gate 1, or Gate 4, respectively. If the dolphins were observed on the "haul outs" near each gate, or engaging people through either of the windows, they were considered preoccupied, and so not a part of that scene.

At the point that a dolphin entered the scene, we assessed its orientation and determined what kind of access it was liable to achieve, if it should remain on its current trajectory, when it passed the gate. We then continued to track that animal to see if there was any change in its orientation or trajectory that resulted in an increase in its access (a "Turn To") by the time it reached the gate. By this scheme, Turn To would include a shift from Binocular to Visio-Echoic, from Monocular to either Binocular or Visio-Echoic, or from None to any type of access. Thus, for every pass, we scored both the type of access the dolphin achieved, and whether it had involved an increase in access relative to the dolphin's original approach.

Within each scene, the time recorded for each animal's pass was the second when the rostrum of that dolphin crossed the far edge of the opening to the gate (i.e., completed its pass). If the animal passed below the gate, we recorded the time of its crossing an imaginary line, extending down from that edge of the gate. We also recorded the identity of every animal in the scene, and whether or not the animals were partnered—that is, swimming parallel and within one body length of another.

Once the time of all such passes were recorded, the relative timing of passes within a scene were assessed. To do this, we classified each dolphin present as "D1" ("Dolphin 1") or "D2" ("Dolphin 2"). A dolphin was classified as D1 if: 1) at least one other animal was in the Vicinity to witness its approach to the gate, and 2) it passed the gate before the next animal did. A dolphin was classified as a D2 if it: 1) was present if/when D1 did a Turn To (and so, could have observed that change), 2) was still present when D1 passed the gate (and so could have observed the access which that animal achieved), and 3) made the next pass of the same gate. Note that, in a given scene, if a third (or any subsequent) animal was also present, a previously passing D2 could then serve as the D1 for that next animal, and so on. In this way, scenes could sometimes be extended to include multiple animals, as long as each, in turn, met the above criteria for D1 and D2. On average, however, only 1.6 animals were present per scene, to potentially witness another animal perform a Turn To.

We will also sometimes refer to any D2 animal as a "Witness". If a Witness was in a position to observe the Turn To of D1, and thereafter did a Turn To itself, that event would be classified as a "gaze follow". If the Witness did not perform a Turn To under those circumstances, that was considered a failure to gaze follow. Since we scored every pass, we could also compare these to the performance of animals who witnessed a pass by D1 that did not involve a Turn To. The main hypothesis that we aim to test here is whether seeing another animal turning to look (or echolocate) through a gate can serve as a cue to promote the next passing animal to likewise Turn To that gate. Thus, in the final analysis, we will compare, within each scene, the likelihood of a Witness doing a Turn To after first observing this behavior in another, compared to D2s who did not first have access to such a change. We cannot, of course, know for certain, in any given instance, if indeed a Witness was attending the D1's behavior. However, given our requirement that an undistracted Witness be close-by enough to observe both any Turn To by D1, as well as D1's pass of the gate, we have tried to make the best case possible, in a naturalistic observational study like this one, for the presumption that one animal could have witnessed and reacted to the actions of another.

A team of seven observers were intensively trained, over several months, to identify all individuals, and to code their access to the gates, as described above. Observers worked in pairs, taking data on the same segments and comparing their results for agreement. Any disagreements or uncertainties were then discussed with the full group, until consensus was reached. If no consensus was achieved, or if animal IDs or types of access were blocked or otherwise unclear, those observations were eliminated from further analysis. Specifically, there were a total of 260 observations in which Identity could not be confirmed, and 209 cases in which type of access could not be confirmed. Once these were removed, there were a total of 7749 passes, upon which we reached consensus, to analyze.

Since our data consist mainly of frequency counts of behaviors that occur during passes, we primarily used Chi-Square analyses to assess and compare them. In particular, for our tests of whether gaze following occurs, and whether partnered animals are more likely to gaze follow than non-partnered, Chi-Square Tests of Independence were used. When we looked at whether the access gained during gaze follows was likely to depend more on the first animal's access, or the overall distribution of access during passes, we used Chi-Square Goodness of Fit tests. When we looked at individual differences, across the seven subjects, in their tendency to gaze follow, and in the distribution of each animal's access, multiple Chi-Square tests were run. These included Bonferroni Corrections, to reduce the likelihood of false positives. And finally, to assess whether the presence of animals in the back habitat could account for apparent gaze follows, a Binomial test was used, with the expectation that if the presence of animals in the back was significantly greater during those events, this could indicate an effect of the back animals on gaze following.

## Results

In this study of 7 bottlenose dolphins, we coded 21 h of activity, in the vicinity of each of two gates that provided access from the animals' main habitat to two rear habitats. On 2218 occasions, individuals were coded as making a change in their trajectory or orientation, as they approached a gate, that increased their access to the back habitats. This represents 28.6% of the total 7749 gate passes observed. Of those *Turn To* changes, 970 were observed by another dolphin (a *Witness*) in the vicinity. *Witnesses* were also present to observe 2635 gate passes by another dolphin that did not include an overt increase in access.

In our first analysis, we tested to see if, after being present to witness a Turn To by the previous animal to pass a gate, the next animal to pass was also more likely to Turn To that gate as well. To do this, we used a 2X2 contingency table in which the rows corresponded to Dolphin 1 (the first to pass the gate) either turning to, or not turning to, the gate as it passed, and the columns corresponded to Dolphin 2 (the Witness) turning to, or not turning to, on its subsequent pass of the same gate. A Chi-Square Test of Independence showed that witnesses were significantly more likely (*X*^*2*^= 327.39, df = 1, *p* < 0.001) to shift to improve their access to a gate after first observing this behavior in another, compared to animals who did not witness such a change. In all, we observed 460 events that met this criteria for *gaze following.*

To assess individual performance on *gaze following*, which required multiple *Chi-Squared* tests, we used the *Bonferroni Correction (*for seven tests, *alpha* needs to be set at < 0.0071*)* to reduce the likelihood of false positives. Six of the seven animals showed a significant tendency to do a *Turn To* after witnessing that behavior in the previous animal (see Table 1). Only one individual (the unrelated, sub-adult female), who was present in the main habitat much less often than the others, failed to show significance in her tendency to *gaze follow* (*X*^2^ = 4.4528, df = 1, *p*-value = 0.0348). This difference may have been a consequence of her small number of opportunities (*N* = 27), compared to the rest of the animals (see Table 1).

Our next analysis examined the effect of swimming with a partner on the likelihood of gaze following. This analysis was based on the same type of Chi-Square contingency table used in our initial gaze following analysis, but compared only dolphins who were partnered—swimming parallel within one body length of one another—versus those who were not. That is, of the total 970 events in which the first animal was witnessed by the second to Turn To the gate, 228 of those involved partnered animals, while 742 involved non-partners. A Chi-Square Test of Independence showed that partners were significantly more likely to gaze follow than animals who were not partnered (*N* = 144; *X*^*2*^ = 29.598, df = 1, *p* < 0.001). In total, 144 events met the criteria for gaze following between partners in our study. It is worth noting, however, that since, overall, the animals were most often not partnered, 69% (*N* = 316) of all gaze follows were performed by non-partnered animals.

**Fig. 4:**
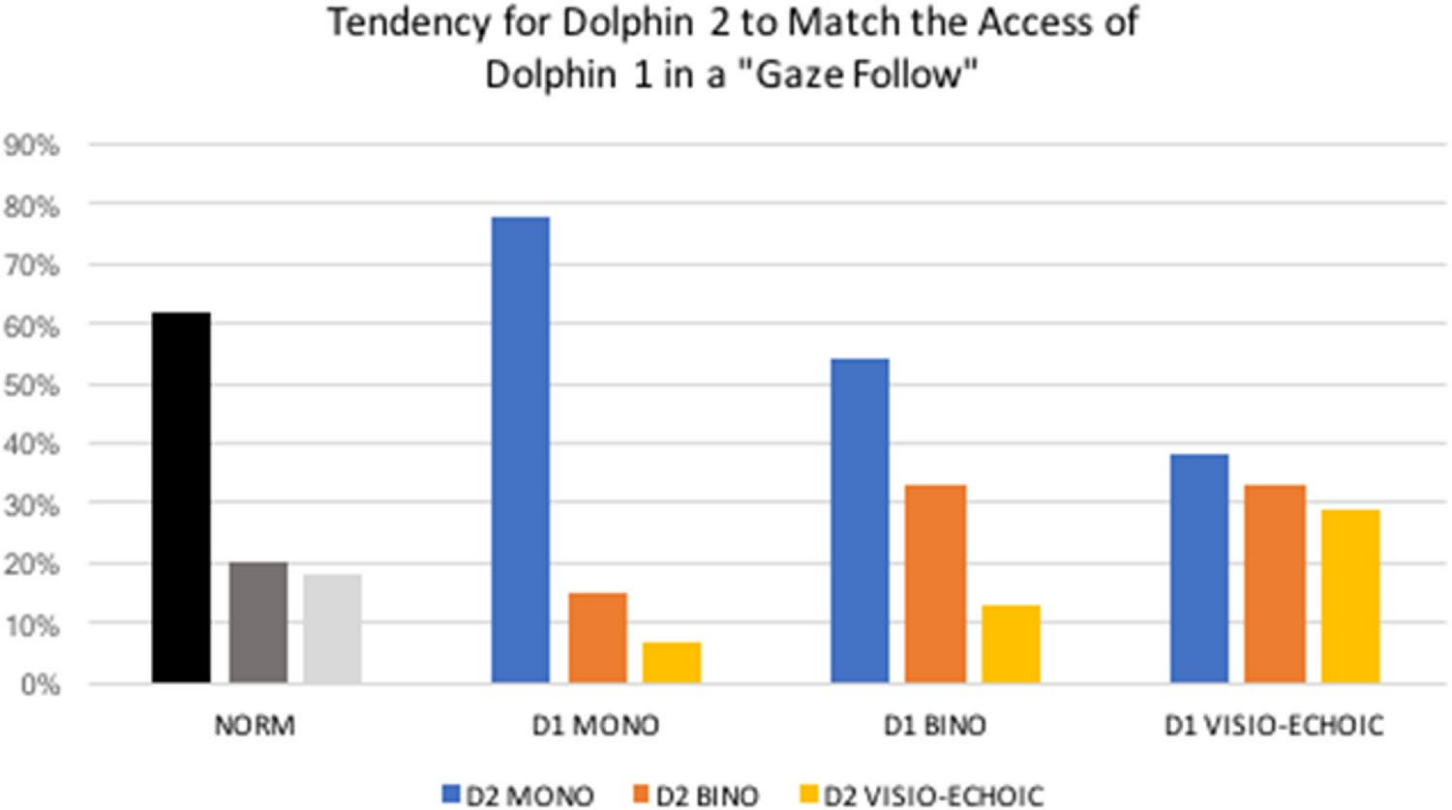
In each cluster, the first bar is the percentage of Turn To events in which the access gained was *Monocular*, the second bar *Binocular*, and the third bar *Visio-Echoic*. The first cluster ("NORM") represents the baseline of access achieved by the first animal to *Turn To* the gate in each scene. The other (colored) clusters are from *gaze following* events only. In the "D1 Mono" cluster, the bars represent the type of access Dolphin 2 achieved after it witnessed Dolphin 1 gain *Monocula*r access. In the "D1 Bino" cluster, the bars represent Dolphin 2's access after it witnessed Dolphin 1 gain *Binocular* access, and in the "D1 Visio-Echoic" cluster, Dolphin 2's access when Dolphin 1 achieved *Visio-Echoic* access

Next we assessed whether, during gaze follows, the type of access achieved by D1 had any influence on the type of access achieved by the Witness, D2. To establish a baseline for the dolphins' general tendency to gain Monocular vs. Binocular vs. Visio-Echoic access, we looked at only the first Turn To in each scene. 923 scenes included at least this one Turn To. (Note that this does not include all Turn To observed, since some scenes included multiple such turns, and we did not want to include any turns that may have been made in response to the activity of others.) Monocular access was achieved on 61.8% (*N* = 570) of these, Binocular was achieved in 20.4% (*N* = 188), and Visio-Echoic in 17.9% (*N* = 165; see Fig. 4). We then compared this distribution to that observed by the *Witness* in all *gaze following* events. That is, we performed *Chi-Square Goodness of Fit* tests comparing the above baseline to D2's access when D1 had achieved *Monocular* access (*X*^*2*^ = 36.833, df = 2, *p* < 0.001), D2's access when D1 had achieved *Binocular* access (*X*^*2*^ = 10.529, df = 2, *p* < 0.005), and D2's access when D1 achieved *Visio-Echoic* access (*X*^*2*^ = 14.863, df = 2, *p* < 0.001; see Fig. 4). In each case, D2 showed a significant tendency to increase its likelihood of producing the type of access it had witnessed D1 achieve.

Finally, it is possible that apparent gaze following events occurred, not because D2 was reacting to D1's Turn To, but because activity in the back habitats had attracted the attention of both animals, individually. To assess this possibility, we examined the likelihood of gaze following occurring when there were, versus were not, any animals present in the back habitat. If animals in the back played a role in D2's response, we would expect their presence to be observed in significantly more than 50% of gaze following events. Of the 460 gaze following events we observed, animals were in the relevant back habitat on 239 occasions, and were absent on the other 221. A Binomial test indicated that the proportion of observations (0.52) in which an animal was in the back during a gaze follow was not significantly different than 0.5 (*p* = 0.428, *NS*), indicating that the Witnesses were unlikely to be reacting to the presence of other dolphins in the back.

## Discussion

In three days of video of seven bottlenose dolphins under human care, we observed 460 instances of conspecific "gaze following". That is, if one animal was observed by another to change its trajectory or posture to give it better access to a back habitat, the witness was also more likely to subsequently re-orient toward that habitat. Animals were more likely to show this effect when swimming in close proximity with a partner. Access could be Monocular, Binocular, or Visio-Echoic, and there was a significant tendency for "gaze followers" to match the type of access gained by the animal they observed. When an animal turned to a back habitat, whether it was "gaze followed" or not, we found no difference in the presence of others in that habitat, minimizing the chance that apparent "gaze following" events were actually cases of animals individually, and thus coincidentally, being drawn to those areas. These findings support adding bottlenose dolphins to the growing list of species that display conspecific "gaze following".

Typically, the first animal to pass a gate would most often attain *Monocular* access, and less frequently *Binocular* or *Visio-Echoic* access. However, during "gaze following", witnesses significantly increased their *Binocular* access when the animal they observed had achieved *Binocular* access, and increased both their Binocular and Visio-Echoic access when the first animal had achieved *Visio-Echoic* access (see Fig. 3). This indicates that the dolphins could discriminate between Monocular, Binocular, and Visio-Echoic access in others. It also suggests that perhaps a greater "show of interest" by the first animal arouses the second animal to achieve a particularly increased level of access as well.

Note that other behavioral cues were also associated with each of these perceptual categories, and thus could have played a role in coordinating those interactions. For example, Visio-Echoic access tended to involve exaggerated head-turns or full body re-orientation through the gates. Binocular access most often involved an overt body rotation, tilting the ventrum toward the gate. Note that the latter could also serve to send a social signal to animals (if any) in the back habitats—also observable by those in the main habitat. That is, dolphins exposing their white underside to another is not only visually salient, but also provides potential access to their genital area (see discussion in Johnson and Norris 1986). Thus, as suggested by Goossens et al. 2008 and Senju and Csibra 2008, it is best for us to recognize that such interactions are mediated by multiple communicative modalities.

These observations also highlight the essentially mimetic nature of "gaze following" behavior in this study. That is, in many—although not all—cases, "gaze following" events involved two animals performing the same posture and trajectory changes, especially when partnered. Dolphins are well documented as proficient mimics (Herman 2002; Kuczaj and Yeater 2006; Jaakola Guarino and Rodriquez 2010). While this may, at first glance, appear to be a confound, it need not undermine the claim that "gaze following" is a distinctive behavior. "Gaze following" does not always depend on behavioral mimicry; the unpartnered animals in our study can make very different approaches to a gate, and still both achieve an increase in access. Plus, the finding that the dolphins tended to ultimately match their type of access helps to make the case that "gaze following" is specifically influenced by the nature of the perceptual access provided. Nonetheless, there may be social functions of gaze following that are similar to other forms of imitation. That is, participating in joint activities, including displaying shared interest, is often included in ethological accounts of development, acclimation, and bonding in social animals (For discussion, see Over and Carpenter 2013; Meltzoff and Williamson, 2013; Johnson 2015).

In this paper, we have made an effort to be cautious in the labels we apply to these behaviors. Echolocation, as described above, requires that an animal point its head directly at its target to get its most discriminative view (Au 1993; Branstetter et al. 2012). It is possible that some of the more than 100 cases observed, in which both animals moved to orient their heads directly toward a back habitat, may be more accurately described as sonar "beam following" events. Acoustic recordings, synchronized with such video, would of course be required to determine if "beam following" actually occurs. Furthermore, the fact that the animals can steer their beams off-center, without changing the direction of their heads, reminds us that head orientation is only a proxy for acoustic access. As a result, we thought it prudent to include quotation marks whenever we used the term "gaze following" for the behaviors we observed in this study.

Our decision to use the term "access" reflects another constraint on the interpretation of these behaviors. The general assumption in gaze following studies is that actions like head-turns indicate a change in attention, and, further, that information along the achieved line-of-sight is being processed by the orienting animal. While this assumption is reasonable enough, it is perhaps more subject to challenge when assessing behavior in an animal with laterally positioned eyes. That is, whenever a dolphin swims directly past a gate, it *may* be visually attending that gate with one eye, or its visual attention may be elsewhere. This could be particularly true for animals, like dolphins, whose eyes are independently controlled (Dawson 1980). Without some direct measure of whether information, available from their orientation, was processed by our subjects, we cannot claim, with certainty, that the animals were indeed *attending* the back habitats. (For a few intriguing gaze following studies that do directly measure the processing of information along the line-of-sight, see Okamoto-Barth et al. 2007; MacLean and Hare 2012). But, by requiring, in our operational definition of "gaze following", that the dolphins make an effortful change in their posture or trajectory to attain an increase in access, we aimed to bolster the assumption that the events analyzed here do generally involve shifts of attention. Nonetheless, we chose to label these behaviors as changes in "access", rather than "attention", to keep us all mindful of the inherent uncertainty involved.

One final issue that arises with any study of following, when more than two animals are involved, is the problem of deciding who is actually following who. Consider the case, with animals A, B, and C, all of whom witnessed each other's movements, when A moves first, then B, then C. How do we resolve whether both B and C were following A, or whether C was following B instead? Sometimes there can be other cues—in timing, signaling, relationship, etc.—that can help to disambiguate such cases. But, especially in long chains of following behavior, this may amount to an ultimately unresolvable problem. In our case, chains of following behavior were relatively short (with, on average, only 1.6 animals present to witness any other individual's change in access). As a result, we opted to focus our analyses on whether only the *next* witness to pass a gate was likely to also increase its access, as the most conservative approach.

But note that this approach would miss cases where the next animal did not gaze follow, but the one after that, also a witness, did! However, adopting an analysis in which *any* witness can gaze follow then raises the original who-is-following-who problem. That is, if C can count as gaze following A, in those cases when B does not gaze follow, then it would be inconsistent *not* to count C as following A in the cases when B *does* follow A. But this, then, would raise the issue of "double counting" C's move, since C would be counted as gaze following both A and B. Plus, again, this also leaves unaddressed the possibility that C was only following B. Fortunately, this was not as great an issue for us, because of the relatively few animals involved in our interactions, and because even by using the "next-animal-to-pass" criterion, which potentially under-estimated the total occurrences of "gaze following" in our corpus, we were able to reach remarkable levels of significance. Nonetheless, these issues are worth considering whenever naturalistic observations of multiple subjects are being used to assess any sort of following behavior.
